# Focused Ethnography in Gerontological Research and Education

**DOI:** 10.1093/geroni/igaa057.041

**Published:** 2020-12-16

**Authors:** Susan Patton

**Affiliations:** University of Arkansas, Fayetteville, Arkansas, United States

## Abstract

Gerontological researchers often use qualitative methods to understand beliefs, values, and shared practices. Focused ethnography (FE) has emerged as a form of qualitative research focused on a specific issue or shared experiences in sub-cultures in specific settings, such as older adults experiencing chronic illness. However, examples of FE in gerontological research literature are few and there is limited methodological guidance on using FE. This presentation provides descriptions of FE based on existing literature and experience in ethnographic research. A systematic review of extant literature was conducted using CINAHL Complete, MEDLINE Complete (Ebsco), and PubMed. Published studies using FE are summarized to demonstrate the methodological foundation and the versatility of FE in exploring health issues in different populations and specific groups of older adults. Nursing students completing a course in chronic illness were trained to conduct an FE on one of the patients in their care. Students were provided a semi-structured interview guide based on the eight questions in Kleinman’s Patient’s Explanatory Model of Illness. The focused ethnographies were then analyzed by qualitative content analysis. Beliefs older adults hold about their chronic illness fell into three categories: regretting lifestyle choices, grieving loss of self, and finding a “new normal”. Focused ethnography is a relevant research method that can be used to understand health behavior of older adults and can be used to teach and evaluate cultural competency in nursing students.

